# SnO_2_/TiO_2_ Thin Film n-n Heterostructures of Improved Sensitivity to NO_2_

**DOI:** 10.3390/s20236830

**Published:** 2020-11-29

**Authors:** Piotr Nowak, Wojciech Maziarz, Artur Rydosz, Kazimierz Kowalski, Magdalena Ziąbka, Katarzyna Zakrzewska

**Affiliations:** 1 Faculty of Computer Science, Electronics and Telecommunications, AGH University of Science and Technology, Al. Mickiewicza 30, 30-059 Kraków, Poland; maziarz@agh.edu.pl (W.M.); rydosz@agh.edu.pl (A.R.); zak@agh.edu.pl (K.Z.); 2 Faculty of Metals Engineering and Industrial Computer Science, AGH University of Science and Technology, Al. Mickiewicza 30, 30-059 Kraków, Poland; kazimierz.kowalski@agh.edu.pl; 3 Faculty of Materials Science and Ceramics, AGH University of Science and Technology, Al. Mickiewicza 30, 30-059 Kraków, Poland; ziabka@agh.edu.pl

**Keywords:** gas sensors, SnO_2_, TiO_2_, thin films, Langmuir-Blodgett technique

## Abstract

Thin-film n-n nanoheterostructures of SnO_2_/TiO_2_, highly sensitive to NO_2_, were obtained in a two-step process: (i) magnetron sputtering, MS followed by (ii) Langmuir-Blodgett, L–B, technique. Thick (200 nm) SnO_2_ base layers were deposited by MS and subsequently overcoated with a thin and discontinuous TiO_2_ film by means of L–B. Rutile nanopowder spread over the ethanol/chloroform/water formed a suspension, which was used as a source in L–B method. The morphology, crystallographic and electronic properties of the prepared sensors were studied by scanning electron microscopy, SEM, X-ray diffraction, XRD in glancing incidence geometry, GID, X-ray photoemission spectroscopy, XPS, and uv-vis-nir spectrophotometry, respectively. It was found that amorphous SnO_2_ films responded to relatively low concentrations of NO_2_ of about 200 ppb. A change of more than two orders of magnitude in the electrical resistivity upon exposure to NO_2_ was further enhanced in SnO_2_/TiO_2_ n-n nanoheterostructures. The best sensor responses R_NO2_/R_0_ were obtained at the lowest operating temperatures of about 120 °C, which is typical for nanomaterials. Response (recovery) times to 400 ppb NO_2_ were determined as a function of the operating temperature and indicated a significant decrease from 62 (42) s at 123 °C to 12 (19) s at 385 °C A much smaller sensitivity to H_2_ was observed, which might be advantageous for selective detection of nitrogen oxides. The influence of humidity on the NO_2_ response was demonstrated to be significantly below 150 °C and systematically decreased upon increase in the operating temperature up to 400 °C.

## 1. Introduction

Nitrogen dioxide (NO_2_) is a highly reactive, hazardous gas and a prominent air pollutant. Despite the fact that only very high concentrations of NO_2_ cause immediate effects: mild irritation of the nose and throat (10–20 ppm), swelling leading to pneumonia or bronchitis (25–50 ppm), and death due to suffocation (above 100 ppm) [1], a prolonged exposure to low amounts of NO_2_ (even of hundreds ppb) may cause breathing problems, including airway inflammation of healthy people and respiratory inefficiency for those with asthma. The threshold limit value (TLV) was set to 3 ppm as time-weighted average (TWA), and 5 ppm as short-term exposure limit (STEL) [2]. Low-threshold, highly sensitive and selective detection of NO_2_ has recently appeared as a particularly important issue due to an increased global conscience of its detrimental influence on the environment [3,4]. Quite drastic measures taken in the case of indoor monitoring of NO_2_ in car interiors [5,6] related to the application of catalysts mounted in the automotive exhaust systems has driven the research towards new concepts of accumulative-type sensors [7].

Metal oxide semiconductors, MOS, such as SnO_2_ and TiO_2_ have been most frequently used as CO and H_2_ gas sensors of the resistive-type [8,9,10,11,12,13,14,15]. Applications of these n-type semiconductors to oxidizing gases are relatively scarce as the resulting high resistance is often beyond the measurement limit. Recently, it has been recognized that it is possible to construct efficient NO_2_ sensors based on Al doped SnO_2_ able to operate properly even under the humidity background [16].

A literature review of SnO_2_-based sensing materials for detection of NO_2_ synthesized by various physical and chemical methods is given in Table 1. From the data included in this table, one can conclude that the efforts are mainly focused on near to room-operating temperature and low NO_2_ threshold. Depending on the composition of the sensing material, even extremely high responses corresponding to the electrical resistance change up to 4 orders of magnitude were demonstrated [17].

All these efforts indicate that the development of a stable and selective NO_2_ gas sensor being capable of fast and accurate detection of extremely low NO_2_ concentrations at near to room temperature is still of prime importance for environmental monitoring, public health and automotive applications.

Recently, a dramatically increased number of publications dealing with p-n and n-n heterostructures as a promising solution to gas sensitivity improvement has been observed [32,37,38,39,40,41,42,43,44,45,46]. In particular, TiO_2_-SnO_2_ n-n heterostructures have been proposed as gas sensitive materials [38,39,45,46].

In 2010, Zeng et al. [39] explained the mechanism responsible for an improved sensitivity of n-n heterostructures. Results from independent experiments showed that the conduction (CB) and valence (VB) band edges of TiO_2_ are above those corresponding to SnO_2_. Therefore, when physical connection is made between TiO_2_ and SnO_2_ grains, a contact potential difference is established which is responsible for an electron transfer from CB of TiO_2_ to CB of SnO_2_. Then, the oxygen pre-adsorption at the surface of SnO_2_ grains is enhanced due to electronic charge injection. Increased concentration of adsorption sites for oxygen is treated as a decisive factor for the observed improvement of the sensing behavior of SnO_2_ with a small addition of TiO_2_ [38,47].

To date, our research performed on TiO_2_–SnO_2_ was related to the gas sensors based on solid-solutions and nanocomposites being a simple mixture of two constituents [38,46,47,48]. Thin films in a form of bi-layers have been studied with much smaller success [45] as the interfaces are usually flat, resulting in much lower surface-to-volume ratio. In order to benefit from both a planar geometry with well defined interfaces between layers and an increased surface-to-volume ratio, a combination of two methods—magnetron sputtering MS and the Langmuir-Blodgett technique—has been proposed in the present work. This innovative approach is expected to yield enhanced responses, particularly to oxidizing gases.

Recently, numerous attempts to use the Langmuir-Blodgett (L–B) technique to grow metal oxide thin films have been reported [49,50,51,52,53,54]. In contrast to Physical Vapour Deposition methods, PVD, [55,56,57,58,59], Langmuir-Blodgett is a non-destructive technique which does not change the structure of the substrate. However, it can affect its electrical properties. Moreover, the L–B deposition is carried out at room temperature and at a normal pressure.

Here, a non-destructive modification of SnO_2_ layer (obtained via magnetron sputtering) by a thin film of TiO_2_ deposited using L–B method is proposed for the construction of an efficient NO_2_ sensor. To the best of our knowledge, this is the first report of application of such combination of methods in the development of SnO_2_/TiO_2_ thin film n-n heterostructures for NO_2_ detection at low concentration and temperature.

## 2. Materials and Methods

### 2.1. Sample Preparation

#### 2.1.1. SnO_2_ Thin Films

Thin films of SnO_2_ were deposited by magnetron sputtering from a metallic Sn target onto corundum CC2.S type supports (BVT Technologies, Czech Republic) dedicated to sensor measurements, silicon and amorphous silica a-SiO_2_ substrates to study their morphological, structural and electronic properties. Reactive sputtering was performed at 50 W, in Ar + 20% O_2_ atmosphere, with base and working pressures of 1.0 × 10^−5^ mbar and 2.0 × 10^−2^ mbar, respectively. Two types of SnO_2_ samples—a-SnO_2_ and c-SnO_2_—were prepared at a substrate temperature of 180 °C during 30 min of sputtering and at 200 °C during 120 min, respectively.

#### 2.1.2. TiO_2_ Thin Films

TiO_2_ thin films were deposited by the L–B method on previously grown a-SnO_2_ thin films to form SnO_2_/TiO_2_ n-n nanoheterostructures. Morevoer, TiO_2_ single layers were obtained on silicon and amorphous silica substrates for XRD, XPS, SEM and optical characterisation.

**Figure 1:**
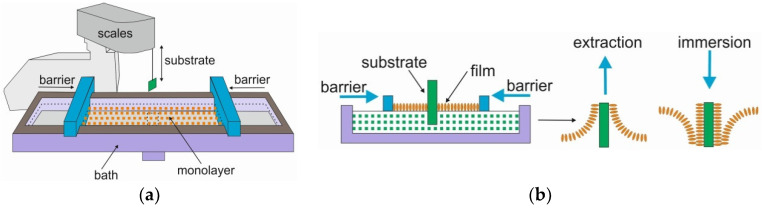
Langmuir–Blodgett deposition technique: (**a**) the setup, (**b**) the method idea.

Langmuir trough and the idea of L–B technique are demonstrated in Figure 1. The experimental set-up consisted of KSV NIMA bath with 270 cm^2^ total area by Biolin Scientific, placed on an anti-vibrational table (Figure 1a). Commercial TiO_2_ rutile nanopowder (Sigma-Aldrich, St. Louis, MO, USA) with a specific surface area, SSA, of 140 m^2^/g was spread on the surface of subphase, i.e., the deionized water with a conductance less than 0.08 μS/cm and rapidly evaporating solvent composed of chloroform:ethanol with 4:1 *v/v* and a typical concentration of 0.5 mg/mL. After spreading, 10 min was allowed for the solvent to evaporate, leaving a loosely packed TiO_2_ layer which was subsequently compressed to a certain surface tension by barriers moving with the speed of 2 mm/min. The surface tension was measured with an accuracy of ±0.1 mN/m using a Wilhelmy plate made of chromatographic paper (Whatman, Piscataway, NJ, USA).

Thin (60 nm) layers of TiO_2_ were obtained by a transfer from the liquid–solid interface to the solid substrates by consecutive immersion and extraction, as shown in Figure 1b. All experiments were performed at room temperature.

**Figure 2:**
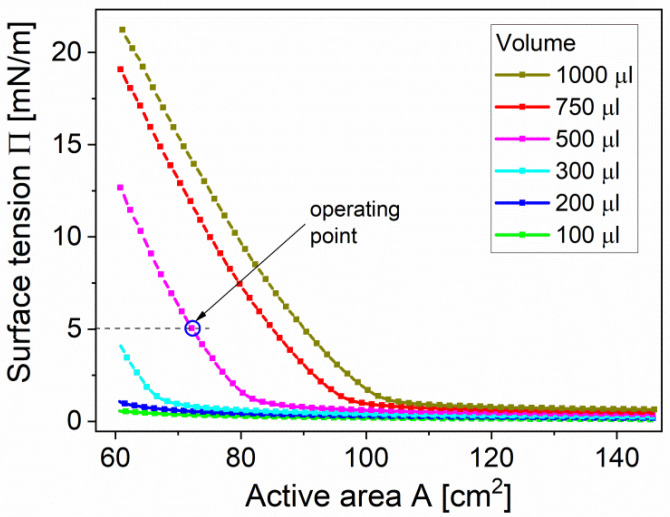
The surface tension vs. active inter-barrier area (Π-A Langmuir isotherms) during deposition of TiO_2_ thin film by L–B.

Prior to this transfer, Langmuir isotherms of suspensions of TiO_2_ rutile nanopowders spread on the surface of water in the Langmuir through were recorded. The surface tension (Π) as a function of total area between barriers (*A*) for different volumes (*V*) of spread TiO_2_ suspension (100–1000 μL) is presented in Figure 2.

The isotherms reveal negligible changes of Π at large *A* until the film is compressed at about 110 cm^2^. Below *A* = 110 cm^2^ for volume *V* of spread TiO_2_ suspension between 100 μL to 1000 μL the surface tension rises sharply until completely compressed in the trough at *A* = 60 cm^2^. The point of rapid growth of value is strongly dependent on *V*. The highest Π equals to 22 mN/m as recorded at *V* = 1000 μL. Even for high *V* values no collapse is observed, thus TiO_2_ layers are stable.

Finally, the TiO_2_ layers were deposited onto the bare substrates or SnO_2_ covered ones at the surface tension Π = 5 mN/m and spread volume *V* = 500 μL.

### 2.2. Characterization Methods

The properties of the prepared thin films were investigated with the use of X-ray diffraction, XRD, scanning electron microscopy, SEM, X-ray photoelectron spectroscopy, XPS, and optical methods.

X-ray diffraction, XRD at grazing incidence GID allowed us to determine the crystal structure of the deposited films. Philips X’Pert Pro diffractometer with CuKα X-ray radiation with wavelength λ = 0.154056 nm, at the incidence angle ω = 3°, was used. The crystallite size, *D*, was calculated from the Debye–Scherrer’s formula given by

(1) $$D=\frac{k\lambda }{\beta \cos\theta }$$

where *k* = 0.9, *β* is the full width at half maximum (FWHM) of a diffraction peak and *θ* is half of the angle at which a given diffraction peak occurs.

The morphology of SnO_2_ and TiO_2_ thin films grown on Si substrates was studied with a NOVA NANO SEM 200 (FEI) Scanning Electron Microscope SEM. The FEI Helios NanoLab 600i Scanning Electron Microscope was applied for the studies of growth of TiO_2_ films on SnO_2_ supports. A chemical analysis of elements was performed by means of Energy Dispersive Spectroscopy, EDS with the latter microscope.

X-ray Photoelectron Spectroscopy (XPS) was employed to assess the surface properties of deposited films. Experiments were performed with the VSW (Vacuum Systems Workshop Ltd., Crowborough, England) instrument working at Kα Mg (1253.6 eV) X-ray radiation and equipped with a concentric hemispherical electron analyser, the details of which are given in [37]. For the calibration of the binding energy BE scale, it was assumed that the position of C 1s line of the adventitious carbon, corresponding to the C–H bond, was equal to 284.6 eV.

Optical spectra of the transmittance T and reflectance R coefficients over a wide wavelength range (220–2200 nm) corresponding to uv/vis/nir regions were taken with the help of Perkin Elmer Lambda 19 double beam spectrophotometer.

**Figure 3:**
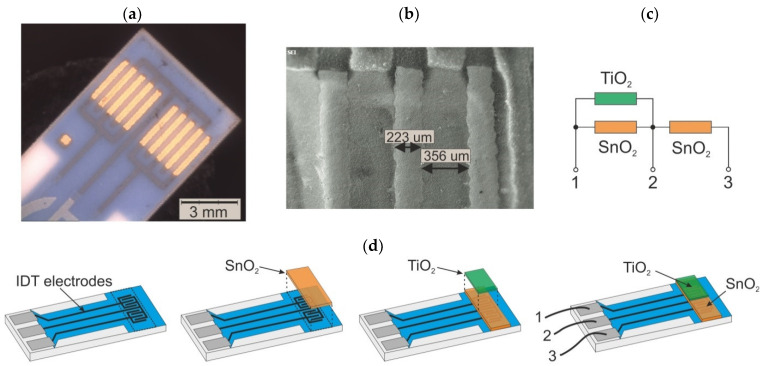
Gas sensor used in experiment: (**a**) SEM photograph of substrate with interdigital Au electrodes and (**b**) electrodes in magnification, (**c**) simplified equivalent circuit of the sensor, and (**d**) successive steps of gas sensor preparation, with sputtered SnO_2_ layer and TiO_2_ thin film deposited by the L–B technique.

The sensors’ responses were measured in a custom-made setup similar to that described elsewhere [60]. The measurements were performed on films deposited onto the final sensors’ platforms, containing double IDT electrodes made of Au and Pt alloy, presented in Figure 3. Due to the double set of electrodes, it was possible to carry out simultaneous studies in both channels and compare the results of SnO_2_/TiO_2_ with those of SnO_2_ under the same conditions.

**Figure 4:**
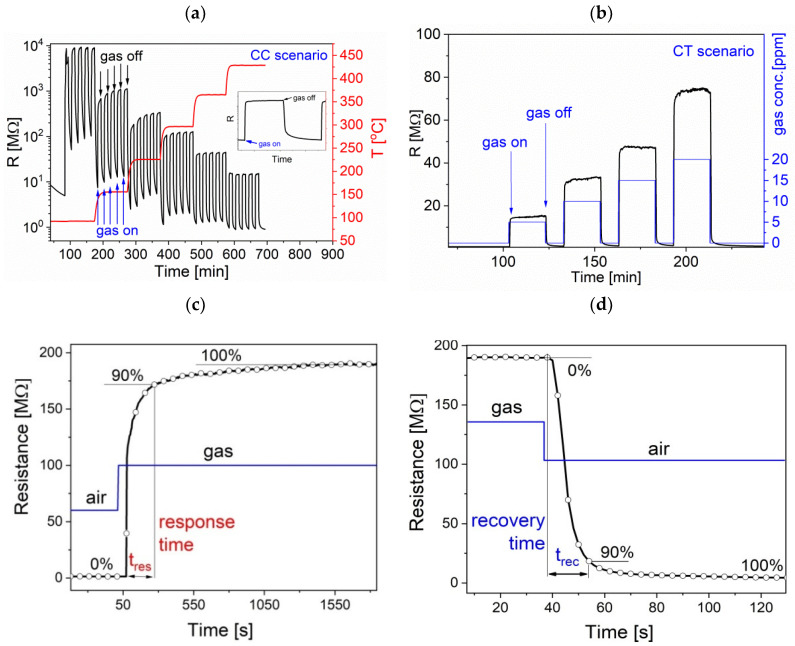
**Gas** sensor response measurements scheme according to (**a**) CC (constant concentration) and (**b**) CT (constant temperature) scenarios, and definitions of (**c**) response t_res_ and (**d**) recovery t_rec_ times.

The sensors were installed in a gas chamber with volume ≈ 30 cm^3^ on a heated workholder. The sensor temperature was determined with a Pt100 thermometer and Agilent 34970A digital multimeter. As the sensor resistance changed over few orders of magnitude, Keithley 6517 electrometer, which sourced a constant voltage U in the range of 1–10 V, was used for the measurement of sensor responses. When the current drawn by the sensor was measured, the sensor resistance was calculated. The samples were collected every 2 s with the use of LabVIEW application working on a PC computer. It controlled the system devices over IEEE 488 (GPIB) bus with the use of SCPI language. The desired gas atmosphere was prepared in a gas system comprised of bottles with synthetic air, NO_2_, H_2_, bubbler for humidifying purposes, and MKS Instruments mass flowmeters controlled with a custom-made mass flow and humidity controller. The sensors were exposed to hydrogen (H_2_, 1000 ppm in a bottle) and nitrogen dioxide (NO_2_, 100 ppm in a bottle), supplied by Air Products, Poland. The requested gas concentration was achieved by controlling the ratio of gas to air flow rate, while the humidity was set up by varying the ratio of dry to humidified air. A total gas flow of 500 cm^3^/min was kept constant during the whole measurement cycle. Prior to performing measurements, the sensor response was stabilized in a synthetic air under pre-set conditions (constant humidity, elevated temperature and chosen gas flow rate), following standard conditioning procedure. Then, the sensor response was measured using two scenarios. In the first one (CC-constant concentration), the sensor was exposed to a series of on/off NO_2_ pulses of the same concentration, with the temperature rising in well defined steps, as shown in Figure 4a. In the second scenario (CT-constant temperature), a series of on/off NO_2_ pulses of increasing concentrations was applied, while the temperature was kept constant (Figure 4b). From the measured sensor responses, one could calculate the basic sensor parameters: response, *S*, response time, *t_res_*, and recovery time, *t_rec_*. The sensor responses, *S*, were defined differently for reducing (H_2_) and oxidizing (NO_2_) gases. In order to obtain *S* higher than 1, S_H2_ = R_0_/R_H2_ was taken as the electrical resistance in air R_0_ divided by that in hydrogen R_H2_, while the inverse ratio was used for NO_2_, i.e., S_NO2_ = R_NO2_/R_0_.

The *t*_res_ and *t*_rec_ parameters were set up as a time required to change the electrical resistance by 90% from the base resistance measured in air (for *t*_res_) or gas (for *t*_rec_) to the stable signal value obtained after the gas or air were introduced, as shown in Figure 4c,d, respectively.

## 3. Results and Discussion

### 3.1. Film Characterization

**Figure 5:**
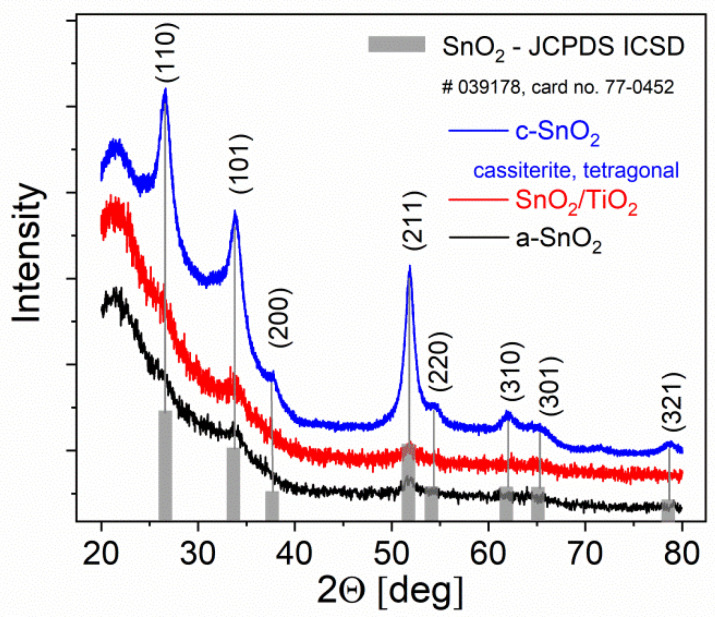
X-ray of diffraction, XRD, patterns of cassiterite, tetragonal SnO_2_ phase, c-SnO_2_, amorphous a-SnO_2_ and SnO_2_/TiO_2_ heterostructured thin films.

Glancing incidence GID X-ray diffraction XRD patterns of the most representative samples prepared in this work are shown in Figure 5. Single SnO_2_ layers deposited by reactive magnetron sputtering on a-SiO_2_ substrate belong to two classes: weakly crystallized almost amorphous a-SnO_2_ and crystalline c-SnO_2_. This difference in the level of crystallization is due to the intentionally changed temperature and sputtering time. XRD pattern of SnO_2_/TiO_2_ bi-layer, composed of a-SnO_2_ thin film grown by magnetron sputtering with TiO_2_ deposited on top of it by the Langmuir–Blodgett method, displays no additional peaks due to TiO_2_ probably because of a small amount of this phase. Tetragonal tin dioxide cassiterite polymorphic form has been confirmed in c-SnO_2_ samples by identification of the most prominent X-ray diffraction lines of crystallographic planes of (110), (101) and (211) with the reference JCPDS data of the card no. 77-0452 (ICSD #039178).

The average crystallite size of c-SnO_2_ thin film was estimated from the Equation (1) as 14 nm. Very weak and wide (101) and (211) diffraction peaks in XRD patterns of a-SnO_2_ indicate that the crystallite size falls below 10 nm, thus the size effect in optical, electronic and sensory properties can be expected.

**Figure 6:**
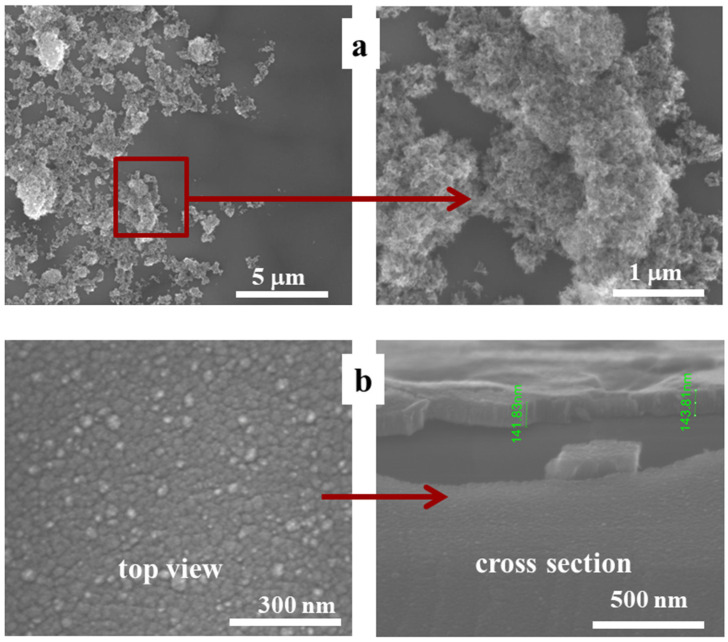
SEM images of thin films on Si substrate: (**a**) TiO_2_, and (**b**) a-SnO_2_.

**Figure 7:**
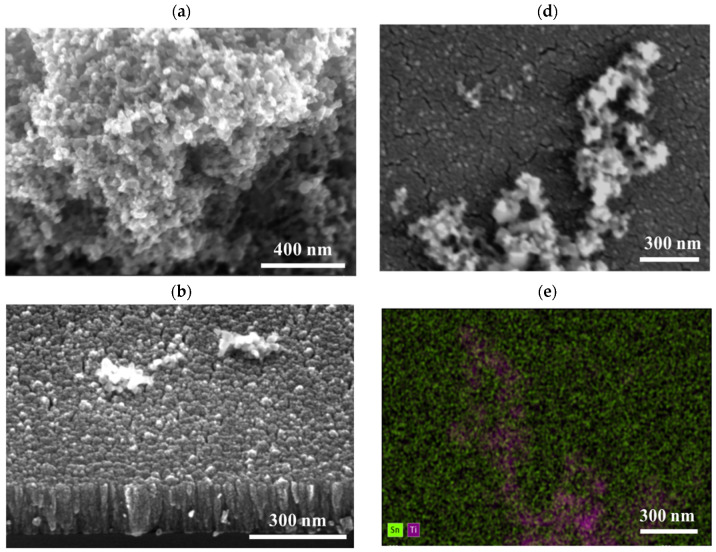

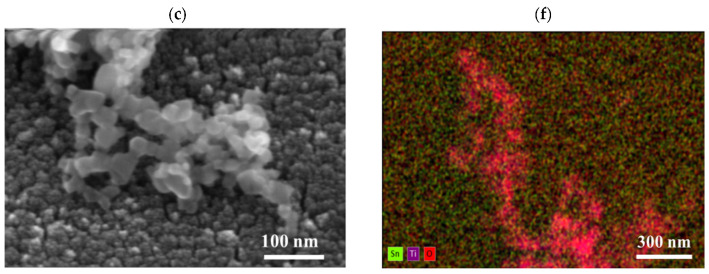
SEM images for TiO_2_ grown by the Langmuir–Blodgett method on the surface of a-SnO_2_ thin films deposited by magnetron sputtering MS: cross sections (**a**,**b**), top view (**c**,**d**) and EDS maps of Sn, Ti, O elements (**e**,**f**).

The morphology of TiO_2_ synthesized by L–B method directly on silicon substrate and that of a-SnO_2_ deposited by MS was investigated by SEM as shown in Figure 6. Figure 7 demonstrates SEM results for TiO_2_ L–B layer on a-SnO_2_ support. Top-view images reveal discontinuous layer composed of TiO_2_ agglomerates (Figure 6a and Figure 7c,d), which might result in an increased surface-to-volume ratio (Figure 7a), advantageous from the point of view of gas sensing. A similar morphology has been reported by Choudhary et al. [61] for ultrathin TiO_2_ layers grown by the same technique.

As far as the morphology of SnO_2_ thin films deposited by sputtering is concerned, it is typical to observe a columnar mode of growth [37]. The cross-sectional images presented in Figure 6b and Figure 7b reveal distinct columns of the diameter increasing in the direction towards the surface. The top view (Figure 6b) shows the relatively smooth surface of a-SnO_2_ with much bigger grains of TiO_2_ nanopowder deposited on top of a-SnO_2_ (Figure 7c,d). EDS mapping (Figure 7e,f) combined with the morphological image in Figure 7d confirms that the discontinuous form observed on the surface of a-SnO_2_ is composed of oxidized titanium.

**Figure 8:**
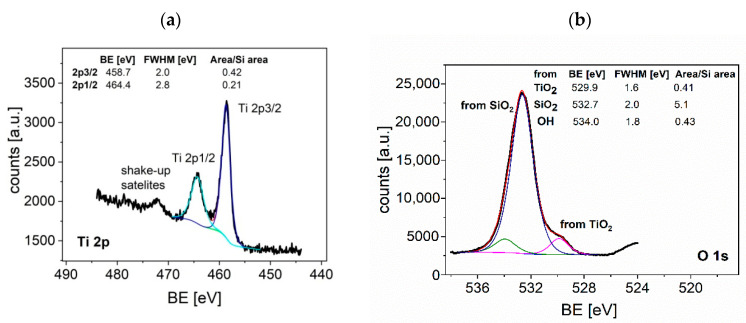

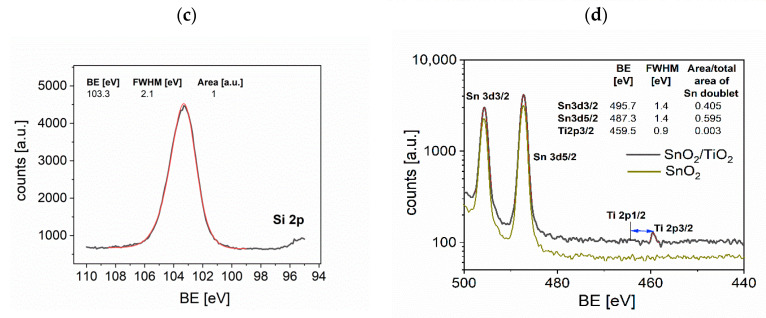
XPS spectra (**a**–**c**) of TiO_2_ thin film deposited on Si substrate by the L–B technique: (**a**) Ti2p, (**b**) O1s, (**c**) Si2p, (**d**) TiO_2_ thin film deposited on SnO_2_ thin film by L–B technique; BE-binding energy.

Further proof of the stoichiometry of the layers deposited by the Langmuir–Blodgett technique has been given by X-ray photoelectron spectroscopy. The oxidation state of Ti ions at the surface of thin films obtained by the L–B method was established by XPS. The Ti2p and O1s spectra are presented in Figure 8a,b, respectively, and the Si peak coming from the substrate is shown in Figure 8c. Moreover, a doublet of Sn 3d_5/2_ and Sn 3d_3/2_ peaks from the a-SnO_2_ layer below can be seen in Figure 8d, supporting the SEM observation of discontinuous growth of TiO_2_. The total area under the Si peak was used as a reference to calculate the relative area of Ti and O peaks. XPS spectra reveal two Ti peaks: Ti2p_3/2_ and Ti2p_1/2_ (at the position 458.7 eV and 464.4 eV, respectively) associated with spin-orbit splitting and assigned to Ti^4+^. Binding energies BE: 487.3 eV and 495.7 eV corresponding to Sn3d_5/2_ and Sn3d_3/2_ XPS peaks, respectively, indicate the presence of Sn^4+^. Recorded O1s XPS peaks are characteristic of the configuration: Ti-O and Si-O (coming from the substrate) with the binding energy 529.9 eV and 532.7 eV, respectively. The O1s peak at 534.0 eV peak can be attributed to –OH groups bounded to the surface of the sample. The O/Ti atomic ratio (calculated by taking into account the respective relative sensitivity factors [62] for the Ti2p_3/2_ peak and O1s peak) is equal to 2.63. The excess of oxygen indicates that the surface of TiO_2_ is over-oxidized as a consequence of adsorption of oxygen, which is important in the first step of gas sensing process.

**Figure 9:**
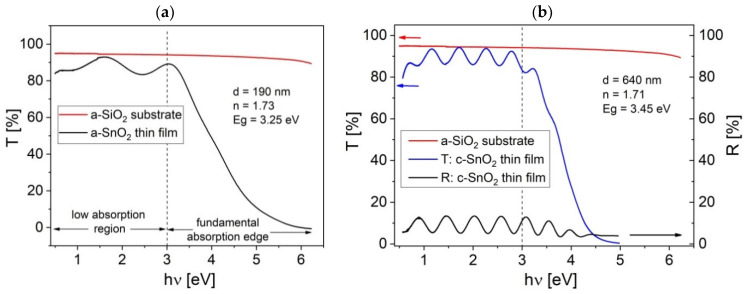
Spectral dependence of the transmittance, T, and reflectance, R, for (**a**) a-SnO_2_, (**b**) c-SnO_2_ thin films; n—calculated refractive index, d—thickness derived from optical spectra; E_g_—band gap.

Figure 9a,b present the transmittance and reflectance spectra of a-SnO_2_ and c-SnO_2_ thin films. The interference fringes of transmittance spectrum over low absorption region enabled us to determine the refractive index, *n*, using the envelope method proposed by Manifacier et al. [63]. Knowing the wavelength positions of extrema in the transmittance spectrum and analysing the amplitude of transmittance coefficient, it is possible to find the film thickness. For a-SnO_2_ sample, refractive index *n* = 1.73 at wavelength *λ* = 700 nm and the film thickness *d* = 190 nm were found.

For c-SnO_2_ film, the transmittance spectrum is richer in extrema because of an increased film thickness d = 640 nm. Moreover, the extrema of the reflectance spectra extend over a wider spectral range than those corresponding to the transmittance. The refractive indices for a-SnO_2_ and c-SnO_2_ thin films are very close, which indicates a similar film density. However, both refractive indices are lower than those usually reported for SnO_2_ (*n* > 2.0) [64]. This may have been caused by the specific columnar growth observed in the SEM cross-sectional images (Figure 6b and Figure 7b). The voids between the columns are probably filled with adsorbed oxygen and water molecules [64].

### 3.2. Gas Sensor Measurements

Gas sensor studies were carried out by following the procedure described in Section 2.2.

**Figure 10:**
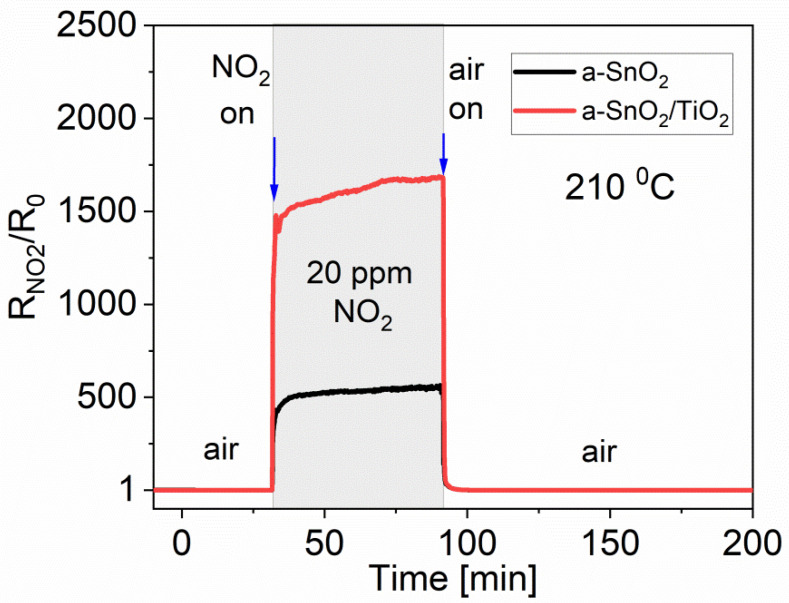
The gas sensor responses, R_NO2_/R_0_, of a-SnO_2_ and SnO_2_/TiO_2_ upon exposure to 20 ppm NO_2_ at 210 °C.

As one can see in Figure 10, both a-SnO_2_ and SnO_2_/TiO_2_ thin film sensors exhibit remarkable R_NO2_/R_0_ responses to 20 ppm NO_2_ at 210 °C of about 500 and 1650, for pure a-SnO_2_, and SnO_2_/TiO_2_ thin films, respectively. Deposition of TiO_2_ onto SnO_2_ improves not only the absolute value of the response but its kinetics as well. Moreover, SnO_2_/TiO_2_ thin films reveal lower resistance in air R_0_, probably due to the injection of electrons from TiO_2_ to SnO_2_, which suggests formation of n-n heterojunction.

**Figure 11:**
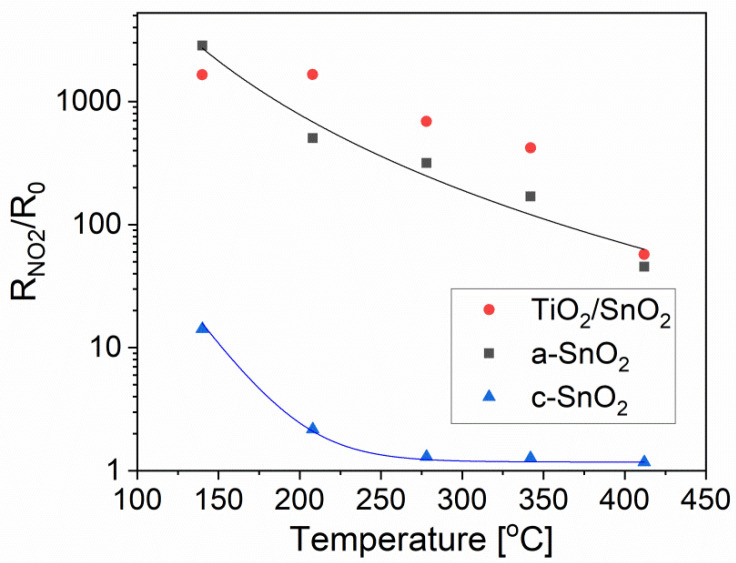
The R_NO2_/R_0_ responses to 20 ppm NO_2_ of a-SnO_2_, c-SnO_2_ and SnO_2_/TiO_2_ thin films vs. operating temperature.

The response, R_NO2_/R_0_, increases systematically when the working temperature decreases, as shown in Figure 11. This indicates a possible interference due to the physisorption of water molecules, which somehow helps in the detection of the oxidizing gas.

**Figure 12:**
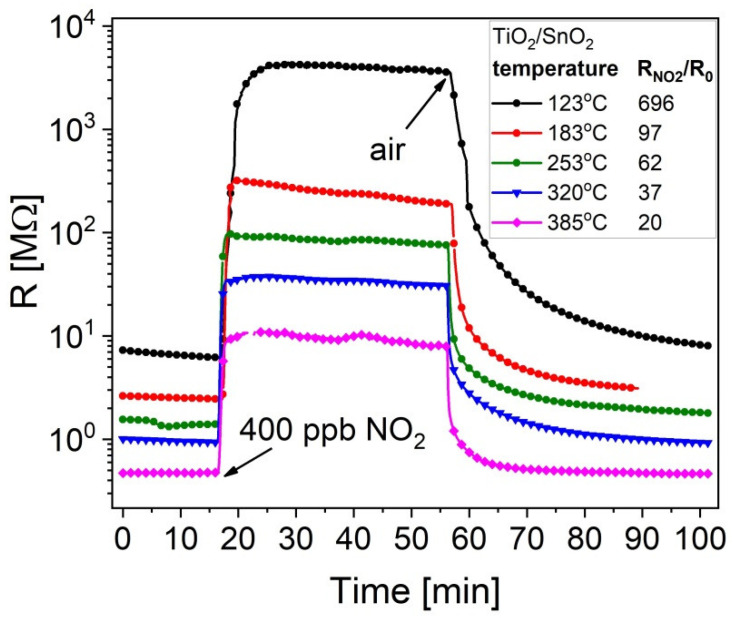
Dynamic changes in the electrical resistance of SnO_2_/TiO_2_ thin films at different operating temperatures, upon exposure to 400 ppb NO_2_.

The same tendency is preserved for lower (400 ppb) concentration of NO_2_ in the case of TiO_2_/SnO_2_ (Figure 12). At 138 °C, the response of a-SnO_2_ to 20 ppm NO_2_ reaches a very high value of about 3000 (Figure 11) while at 123 °C, the response of SnO_2_/TiO_2_ to 400 ppb NO_2_ is as high as 700 (Figure 12). The electrical resistance R_0_ in air decreases systematically with temperature, which is typical for semiconducting behaviour.

Over the temperature range extending from 200 to 350 °C, SnO_2_/TiO_2_ thin film exhibits a higher response than that of a-SnO_2_. As can be seen in Figure 11, within this temperature range, the response R_NO2_/R_0_ is 2.5 to 3 times higher for SnO_2_/TiO_2_ compared with that of a-SnO_2_. It can be seen that amorphous SnO_2_ demonstrates much better sensing properties than crystalline SnO_2_, probably due to the well known size effect [9] but at the same time, one cannot exclude the influence of film thickness on the gas response.

**Figure 13:**
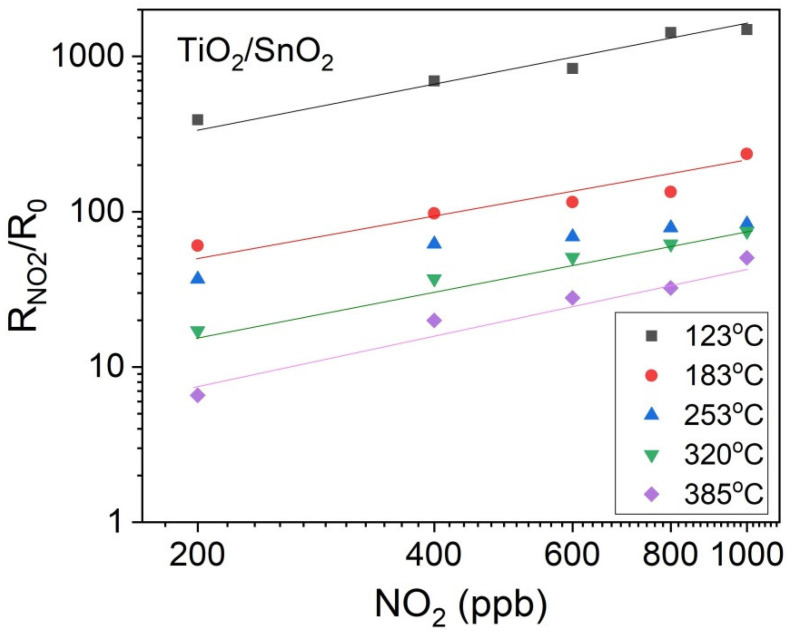
The SnO_2_/TiO_2_ thin film response to low NO_2_ concentrations at different operating temperatures.

Figure 13 illustrates the responses of SnO_2_/TiO_2_ thin films as a function of NO_2_ concentration down to 200 ppb at constant temperatures chosen within the range extending from 120 °C to 400 °C. Bi-layers of SnO_2_/TiO_2_ are sensitive even to 200 ppb NO_2_ with the responses of 390@123 °C and 6.6@385 °C.

Based on the results discussed above, the analysis of kinetics of the response and the signal recovery was performed. As Table 2 shows, both response *t_resp_* and recovery *t_rec_* times gradually decrease with the increasing temperature and gas concentration. At all operating temperatures above 150 °C, *t_resp_* is rather small and equals to about 4–12 s. The SnO_2_/TiO_2_ sensor recovers relatively quickly, *t_rec_* amounts to 9–28 s above 150 °C. Such a fast reaction to NO_2_ and recovery in air are typical for low amounts of NO_2_ and suggest a dominant role of the surface adsorption processes due to the small film thickness.

Small values of *t_resp_* and *t_rec_* along with very good responses, observed at very low NO_2_ concentrations, make such a sensor very promising for environmental applications.

**Figure 14:**
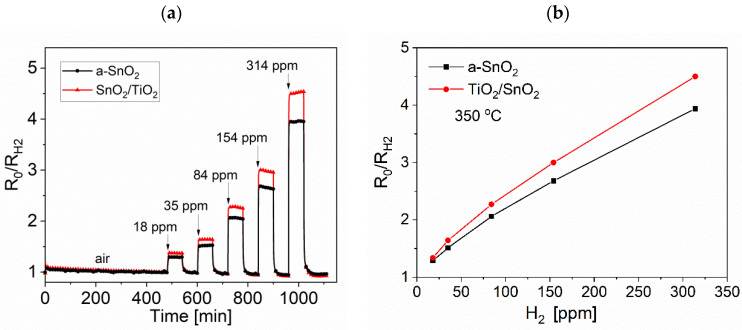
Responses R_0_/R_H2_ of a-SnO_2_ and SnO_2_/TiO_2_ thin films to step changes in H_2_ measured according to scenario CT: (**a**) dynamic characteristics at 350 °C and (**b**) response as a function of H_2_ concentration at 350 °C.

**Figure 15:**
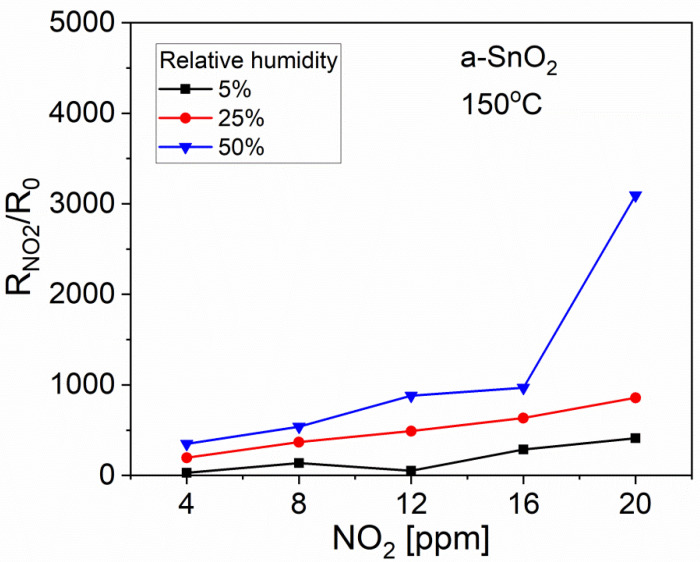
Influence of humidity on the R_NO2_/R_0_ responses of a-SnO_2_ thin films to varying NO_2_ concentrations at a constant operating temperature 150 °C.

The effects of interfering agents, such as a reducing gas and humidity, were taken into account, as demonstrated in Figure 14 and Figure 15, respectively.

The responses of both a-SnO_2_ and SnO_2_/TiO_2_ to the reducing gas H_2_ are much smaller than those to NO_2_. The highest response to H_2_ was observed at 350–400 °C, but even at 314 ppm the S_H2_ = 3.9 for a-SnO_2_ layer and S_H2_ = 4.5 for SnO_2_/TiO_2_ bi-layer. The decoration of SnO_2_ with TiO_2_ thin film improved the response to H_2_ by only 18%.

As mentioned in Section 2.2*,* all the gas sensing measurements were performed at relative humidity RH = 50%. This level of humidity is treated as ‘normal’ because the sensors usually work under such environmental conditions. In order to investigate the influence of humidity on the sensing characteristics, a-SnO_2_ sample was tested also at lower RH values, as shown in Figure 15 and Table 3. One can conclude that a higher humidity improves the response to NO_2_. The effect is the quite pronounced at lower operating temperature of 150 °C. The humidity interference is negligible at higher operating temperatures, which suggests a predominant role of the physisorption of molecular water over the chemisorption of OH^−^ groups [16,65].

## 4. Discussion

SnO_2_ and TiO_2_ are both n-type metal oxide semiconductors. Their sensing mechanism is controlled by the surface phenomena and their resistance changes upon exposure to different gas atmospheres. In the first step of gas sensing, it is usually assumed that oxygen is adsorbed in different atomic or molecular forms, i.e., O^2−^, O^−^, and O_2_^−^ [66,67]. Such behaviour can be expressed by the following reactions (2–5):

O_2(gas)_ → O_2(ads)_ (2)

O_2(ads)_ + e^−^ → O_2_^−^_(ads)_ (3)

O_2_^−^_(ads)_ + e^−^ → 2O^−^_(ads)_ (4)

O^−^_(ads)_ + e^−^ → O^2−^_(ads)_ (5)

The ionized oxygen O_2_^−^ species dominate at temperatures below 150 °C [16], while for temperatures above 150 °C, the predominant oxygen forms are O^‑^ and O^2−^. When the sensor is exposed to pure air atmosphere at a constant temperature, the oxygen molecules adsorb at the sensing material surface, and capture free electrons from the conduction band. As a result, the sensor conductance decreases. After some time, an equilibrium is reached and the sensor resistance stabilizes. After exposure to NO_2_ oxidizing gas, the effect of electron capturing proceeds further (reaction 6). Moreover, NO_2_^−^ ions can be created in reaction with previously adsorbed oxygen ions O^2−^_(ads)_ (reaction 7). Reactions 6 and 7 cause a reduction of the sensor conductance. At the same time, a reverse reaction 8 with NO_2_^−^_(ads)_ losing an electron may take place.

NO_2_ + e^−^ → NO_2_^−^_(ads)_ (6)

NO_2_ + O^2−^_(ads)_ + 2e^−^ →NO_2_^−^_(ads)_ + 2O^−^_(ads)_ (7)

NO_2_^−^_(ads)_ + O^−^_(ads)_ → NO_2(gas)_ + O^2−^_(ads)_ (8)

It should be stressed that reaction with NO_2_ can take place (reaction 6) without the presence of air (and therefore oxygen) because NO_2_ itself contains oxygen and forms an oxidizing agent. Reaction to NO_2_ in an oxygen-free atmosphere is therefore stronger than in an oxygen-rich one, because the oxygen molecules adsorbing at the surface reduce the number of free sites where NO_2_ adsorption can occur.

Tin dioxide, SnO_2_ has been successfully applied to NO_2_ detection, as can be concluded based on the publications listed in Table 1. Santos et al. [18] demonstrated the selective NO_2_ detection of low concentrations down to 100 ppb by thin films of SnO_2_ at 200 °C. The technology of SnO_2_ affects its morphology, which, as a consequence, influences the gas response and its kinetics. The examples of different methods of SnO_2_ synthesis—sol gel [19], chemical spray deposition [24], chemical vapor deposition [25] and vapor phase deposition [11]—were given. Modification of SnO_2_ by other metal oxides such as ZnO, WO_3_, and TiO_2_ was demonstrated as an efficient means to enhance the sensing performance. A very high response (as high as 12800) to 5 ppm NO_2_ at relatively low temperature of 150 °C was reported by Sukunta et al. [17] for heterostructures of SnO_2_ nanoparticles-WO_3_ nanotubes. A specific morphology providing well developed surface is required for an improved gas sensing response. It is believed that the higher surface-to-volume ratio of the sensing material results in an increased density of active centers for chemisorption. Sharma et al. [23] published the results of extended studies on gas sensing with various structures: WO_3_/SnO_2_, TeO_2_/SnO_2_, CuO/SnO_2_, ZnO/SnO_2_ among which one can find TiO_2_ nano-thin micro-clusters loaded over SnO_2_ that manifested a relatively high response of 825 to 10 ppm NO_2_ at a low temperature of 90 °C.

The Langmuir–Blodgett technique is usually applied to form monolayers of amphiphilic molecules. Therefore, in attempts to use this method in gas-sensing applications, organic compounds such as porphyrin [68,69], benzenedicarboxylic acids [70], and polypyrrole [71] have been most frequently employed. Formation of TiO_2_ or SnO_2_ thin films is often associated with using precursors of metal oxides, e.g., titanium alkoxides [72], polianiline-TiO_2_ [73] or complexes of metal salts of fatty acid or amines, e.g., ODA-stannate complexes [74], or ODA-KTiO_2_ [75]. Obtained films are then subject to a thermal decomposition which removes the organic part. Another method is to deposit organic amphiphilic particles with a chemical affinity to TiO_2_ or SnO_2_ derivatives. Then, the previously obtained layer is immersed in the proper solution (e.g., potassium titanium oxalate, PTO [76,77] or SnO_2_ derivatives) and thermally treated. There are few reports on using the L–B method to form thin films directly from crystallites of metal oxides [61,78,79].

Choudhary et al. [61] demonstrated the well developed surface morphology of TiO_2_ layers grown using the Langmuir–Blodgett technique. The aggregation of nanoparticles and surface coverage can be tuned by the target surface pressure. It has been claimed [61] that discontinuous TiO_2_ layers such as those observed in the course of our studies contain large amount of defects that favourably affect the sensor response. The same group from Bhabha Atomic Research Center, Mumbai, India published a series of papers [77,79] on SnO_2_ and SnO_2_-TiO_2_ thin films deposited by L–B method for gas sensing and photoelectrochemical application.

It is well known [65] that as the gas sensors usually operate in the atmosphere containing water, two processes can be responsible for H_2_O surface adsorption:

- physisorption of water in its molecular form that occurs at lower temperatures
- chemisorption of OH^−^ taking place at higher temperatures above 300 °C

However, if both oxygen and water molecules are present in the atmosphere, there is a competitive adsorption between O_2_- and H_2_O-related surface species. As a result, MOS responses to gases are distinctly different under dry and humidified atmospheres [80,81,82].

A decrease in the gas sensor response in the presence of elevated level of humidity is usually explained by a reduction of the effective sensing area. However, the opposite behaviour can also be seen when the detected molecule directly reacts with active hydroxyl groups OH^­^, thus improving the sensor response [16]. In view of the results presented here, it can be assumed that the physisorption of water molecules is probably responsible for an increase in the NO_2_ response as the most pronounced influence of humidity is observed at 150 °C. It is possible that at this relatively low temperature, there is a strong competition between oxygen and water adsorption. The elimination of the adsorption of oxygen species might be beneficial to NO_2_ gas sensing, as already discussed (eq.6) the presence of oxygen is not necessary to form NO_2_^−^ sites at the sensor surface.

## 5. Conclusions

The SnO_2_/TiO_2_ n-n thin film nanoheterostructures were obtained by depositing discontinuous TiO_2_ thin films on previously sputtered thicker SnO_2_ layers using the Langmuir–Blodgett technique with a nanopowder of rutile as a starting material. The morphological, structural and electronic properties of pure SnO_2_ and SnO_2_/TiO_2_ heterostructures and their responses to NO_2_ gases were studied. The most important results of this research can be summarized as follows:

- The heterostructures composed of TiO_2_ agglomerated discontinuous layer on the SnO_2_ thin film with a columnar mode of growth have a higher gas response than pure SnO_2_ for both reducing (H_2_) and oxidizing (NO_2_) gases.
- Amorphous a-SnO_2_ demonstrate a much higher response to NO_2_ than their crystalline counterparts c-SnO_2_, probably because of the size effect.
- SnO_2_/TiO_2_ heterostructures are selective and sensitive even to low concentrations of NO_2_ which can be attributed to the electron injection from the conduction band CB of TiO_2_ to CB of SnO_2_.
- The significant increase in NO_2_ response occurs at an operating temperature below 150 °C where a considerable influence of humidity has been demonstrated; this effect is probably due to the competitive physisorption of water against chemisorption of oxygen and hydroxyl groups.
- SnO_2_/TiO_2_ n-n nanoheterostructures in a form of thin films have proven to be highly sensitive and selective to NO_2_ with a threshold lower than 200 ppb.

## Figures and Tables

**Table 1:** Survey of NO_2_-sensing materials based on SnO_2_ prepared by various physical and chemical methods.

NO_2_-Sensing MOS	Synthesis Method	Operating Temperature	R_NO2_/R_air_	Concentration [ppm]	Reference, Year
SnO_2_	rf-sputtering	200 °C	18	0.1	[18], 1997
SnO_2_	sol-gel	150 °C	72	500	[19], 2007
ZnO–SnO_2_	reversed microemulsion	250 °C	34.5	500	[20], 2008
WO_2_–SnO_2_	sol precipitation	200 °C	186	200	[21], 2010
In_2_O_3_–SnO_2_	co-precipitation	200 °C	7.5	1000	[22], 2006
TiO_2_/SnO_2_	e-beam evaporation	90 °C	825	10	[23], 2013
SnO_2_	chemical spray deposition	350 °C	60	500	[24], 1999
SnO_2_	vapor phase deposition	300 °C	9	0.2	[11], 2005
SnO_2_	chemical vapor deposition	450 °C	0.93	10	[25], 1999
SnO_2_+Bi_2_O_3_	vapor-liquid-solid method	250 °C	56.9	2	[26], 2018
SnO_2_ + graphene SnO_2_+MWCNT	sol–gel method	RT	~9.5 ~4.5	20	[27], 2016
Au/SnO_2_:NiO	sputtering	200 °C	∼185	5	[28], 2019
SnO_2_	spray pyrolysis	150 °C	556	40	[29], 2017
SnO_2_/SnS_2_	high temperature oxidation	80 °C	5	8	[30], 2017
Pd/SnO_2_ Pt/SnO_2_	co-precipitation	30 °C + 7mW uv	3400 1500	5	[31], 2017
SnO-SnO_2_	hydrothermal method	RT	2.5 4.5 15	0.2 1 100	[32], 2018
SnO_2_-WO_3_	thermal decomposition	150 °C	12800	5	[17], 2018
SnO_2_-graphene	hydrothermal method	75 °C	225	0.35	[33], 2019
ZnO+SnO_2_	electrospinning	200 °C	258	100	[34], 2019
SnO_2_@SnS_2_	hydrothermal method	RT, blue light	5.2 57.3	0.2 5	[35], 2020
SnO_2_/ZnO	sputtering	100 °C	67	100	[36], 2020

RT—room temperature, uv—ultraviolet irradiation, MWCNT—multiwall carbon nanotubes; NT—nanotubes.

**Table 2:** Kinetics of the SnO_2_/TiO_2_ thin film response to NO_2_ of two different concentrations; t_resp_—response time, t_rec_—recovery time.

Temperature [°C]	400 ppb NO_2_			2000 ppb NO_2_		
	t_resp_ [s]	t_rec_ [s]	R_NO2_/R_0_	t_resp_ [s]	t_rec_ [s]	R_NO2_/R_0_
123	62	42	696	26	58	847
183	11	22	97	10	9	350
253	10	17	62	4	10	136
320	12	28	37	4	11	101
385	12	19	20	4	-	-

**Table 3:** R_NO2_/R_0_ responses of a-SnO_2_ sensor to 12 ppm NO_2_ at various temperatures and for different relative humidity values.

RH %	R_NO2_/R_0_			
	150 °C	220 °C	235 °C	335 °C
5	51	-	-	13
25	489	135	127	-
50	881	147	128	-
75	-	94	60	-
